# Robot Calibration Sampling Data Optimization Method Based on Improved Robot Observability Metrics and Binary Simulated Annealing Algorithm

**DOI:** 10.3390/s24196171

**Published:** 2024-09-24

**Authors:** Huakun Jia, Hanbo Zeng, Jiyan Zhang, Xiangyang Wang, Yang Lu, Liandong Yu

**Affiliations:** College of Control Science and Engineering, China University of Petroleum (East China), Qingdao 266580, China

**Keywords:** sampling data optimization, calibration, Product of Exponentials model, observability metrics, industrial robot

## Abstract

As the societal demand for precision in industrial robot operations increases, calibration can enhance the end-effector positioning accuracy of robots. Sampling data optimization plays an important role in improving the calibration effect. In this study, a robot calibration sampling point optimization method based on improved robot observability metrics and a Binary Simulated Annealing Algorithm is proposed. Initially, a robot kinematic model based on the Product of Exponentials (POE) model and a generalized error model is established. By utilizing the least squares method, the ideal pose transformation relationship between the robot’s base coordinate system and the laser tracker measurement coordinate system is derived, resulting in an error calibration model based on spatial single points. An improved robot observability metric combined with the Binary Simulated Annealing Algorithm (BSAA) is introduced to optimize the selection of calibration sampling data. Finally, the robot’s parameter errors are obtained using a nonlinear least squares method. Experimental results demonstrate that the average end-effector positioning error of the robot after calibration can be reduced from 0.356 mm to 0.254 mm using this method.

## 1. Introduction

With the increasing demands for production in society, the application of robots in the industrial production field is becoming increasingly widespread. However, errors inevitably arise between the robot’s structure and design parameters during production, assembly, and transportation processes, leading to errors in the robot’s end-effector positioning. Undoubtedly, the existence of these errors poses significant challenges to the development of robot applications. A commonly used solution is to obtain the errors between the actual structural parameters of the robot and the theoretical parameters through parameter calibration to compensate for the robot’s kinematic model and reduce end-effector positioning errors.

Denavit and Hartenberg introduced the classic kinematic model, the DH (Denavit–Hartenberg) model, which uses link lengths, link twist angles, link offsets, and joint angles to describe the pose relationship between adjacent joint links. This model is intuitive and has been used consistently up to the present day [1]. Hayati and others improved upon the DH model by introducing the MDH (Modified Denavit–Hartenberg) model to address the issue of mathematical description when adjacent joint axes are parallel [2]. Brockett first proposed the POE (Product of Exponential) model for robot kinematic modeling [3]. The POE model describes the robot using only the base coordinate system and end-effector coordinate system, significantly reducing the modeling complexity while also satisfying continuity and completeness requirements.

For the same set of structural parameter errors, the end-effector positioning error of a robot will vary depending on its configuration. It is usually desirable to find a configuration that can most effectively “reflect” the structural parameter errors of the robot, thus obtaining more accurate parameter identification results. This process is referred to as robot calibration configuration optimization selection. Directly optimizing the selection of calibration configurations based on the analysis of a robot’s kinematic model is often not easy to achieve. Therefore, in practice, engineers typically first obtain a large amount of calibration sampling data within the robot’s workspace and then optimize the selection of data.

Scholars have proposed various observability metrics for optimizing the selection of robot calibration configurations, among which observability metrics based on the singular values of the Jacobian matrix have been widely applied. Borm and Menq derived an observability metric, $O_{1}$, using the geometric mean of the singular values of the Jacobian matrix [4]. Driels and Pathre defined $O_{2}$ as the ratio of the minimum singular value to the maximum singular value of the Jacobian matrix [5]. Nahivi and Hollerbach defined $O_{3}$ as the minimum singular value of the Jacobian matrix [6]. They also defined $O_{4}$ as the product of $O_{2}$ and $O_{3}$ [7,8]. Sun and Hollerbach defined $O_{5}$ as the reciprocal of the sum of the reciprocals of all singular values of the Jacobian matrix [9]. Wang et al. proposed a more universal evaluation metric, $O_{6}$, which in certain special cases can be considered as $O_{1}$, $O_{3}$, or $O_{5}$ [10].

Joubair et al. evaluated the effectiveness of five commonly used observability metrics in robot calibration. The results indicated that in most cases, $O_{1}$ is the best observability metric [11]. Jia determined the optimal number of calibration data points using $O_{1}$ and, as a constraint, maximized $O_{3}$ to obtain the optimal calibration dataset using an improved DETMAX algorithm [12]. Chen et al. proposed metrics to evaluate the dispersion and uniformity of the distribution of the robot end-effector in the workspace. They combined these metrics with $O_{1}$, $O_{2}$, and $O_{3}$ using a normalization function to derive a comprehensive observability metric and obtained the optimal calibration dataset [13]. Chen et al. combined $O_{1}$ and $O_{3}$ using a dynamic weighted aggregation method to obtain a comprehensive observability metric [14].

Jiang proposed the evaluation index D for identifiable parameter biases and a new index P for assessing the distribution of robot end-effectors. Subsequently, the DETMAX algorithm was utilized to optimize sampling points sequentially by incorporating the indices D, O_1_ and P [15]. Jiang also introduced observability indices to evaluate robot stiffness for sampling point optimization [16]. Additionally, Hu and others similarly presented observability indices for assessing robot stiffness [17]. Stefanie and colleagues implemented optimal measurement pose selection for robots based on optimal experimental design theory [18]. 

Furthermore, there are research efforts that focus on sampling point optimization without using dedicated evaluation indices for this purpose, although such studies are limited. Toquica and collaborators established several sub-workspaces within a robot’s workspace, selecting a certain number of sampling points randomly in each subspace for kinematic parameter identification [19]. The results indicate that partitioning-based calibration methods serve as viable alternatives to traditional calibration methods. Although the final calibration accuracy may not be optimal, this approach saves time and reduces labor costs.

The problem of sampling point optimization can be viewed as a kind of optimization problem. Various metaheuristic algorithms can be employed to address optimization problems, such as particle swarm optimization [20,21], genetic algorithms [22,23], simulated annealing [24,25], etc.

While the aforementioned works have improved the accuracy of robot end-effector positioning, there are still issues with the methods used, including the following: (1) Individual observability metrics can only represent limited meanings, and current comprehensive observability metrics proposed for this problem have not considered the global nature of the robot’s end-effector position. (2) The search algorithms used are prone to becoming stuck in local optimal solutions.

In response to the issues mentioned above, a new observability metric is proposed in this paper. Firstly, a global metric $O_{d}$ is introduced to evaluate the global distribution of the calibration sampling data in space. Subsequently, the arctangent function is used to normalize $O_{1}$, $O_{3}$, and $O_{d}$, establishing a comprehensive observability metric $O_{c}$. This metric is then combined with the Binary Simulated Annealing Algorithm proposed in this study for optimizing calibration sampling data. The robot kinematic parameters calibrated using this method are more accurate, and experiments show a significant reduction in the end-effector positioning error of the robot.

Section 1 introduces the work carried out by other researchers in robot calibration, mainly focusing on optimizing calibration sampling data. Section 2 mainly describes the robot’s kinematic model and error calibration model. Section 3 presents the calibration sampling data optimization selection method proposed in this study, including the comprehensive observability metric $O_{c}$ and the Binary Simulated Annealing Algorithm. Section 4 provides the experimental results. The discussion is presented in Section 5.

## 2. Modeling

This section primarily focuses on the mathematical models related to robots. In Section 2.1, the POE kinematic model of the UR10 robot is introduced, establishing the transformation relationship between the position of the robot end-effector tool center relative to the robot base. In Section 2.2, the robot error calibration model is presented, establishing the relationship between the coordinate errors of the robot end-effector tool center and the errors in the robot’s kinematic parameters. These kinematic parameters encompass the robot’s kinematic parameters and the spatial transformation parameters between the coordinate system of the laser tracker in the calibration system and the robot base coordinate system.

### 2.1. The Robot Kinematic Model Based on the POE Model

**Figure 1:**
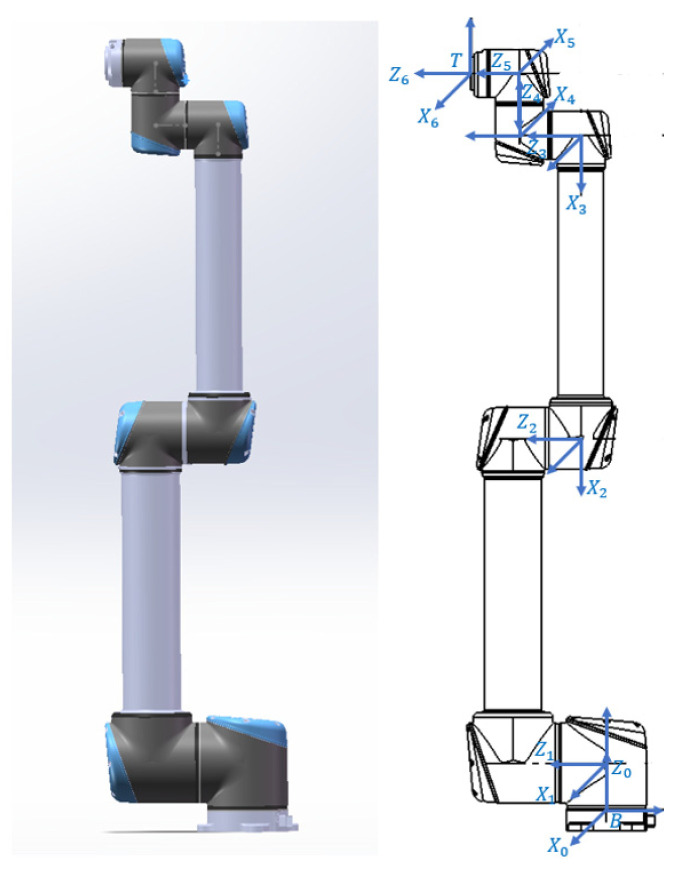
Structure of UR10 robot.

This research focuses on the UR10 robot; the structure is shown in Figure 1. Due to the presence of adjacent joint axes that are parallel (or nearly parallel) in this robot, modeling such robots using the traditional DH model can lead to singularities in the model. Therefore, it is common to consider this by using the M-DH model or the POE model for modeling. The POE model is based on screw theory, and using this model ensures the continuity of the robot model.

According to Chasles’ theorem, the exponential coordinates of motion screws represents the helical motion of a rigid body [26]. For the ith joint, the following can be obtained:

(1) $$e^{{\hat{\xi }}_{i}\theta_{i}}=\left[\begin{array}{cc} e^{{\hat{\omega }}_{i}\theta_{i}} & \left(I-e^{{\hat{\omega }}_{i}\theta_{i}}\right)\left(\omega_{i}\times V_{i}\right)+\theta_{i}\omega_{i}\omega_{i}^{T}V_{i}\\ 0_{1*3} & 1 \end{array}\right]$$

(2) $$V_{i}=\omega_{i}\times q_{i}$$

where $\omega_{i}={\left[\omega_{1i},\omega_{2i},\omega_{3i}\right]}^{T}$ is the direction vector of the axis of the ith joint of the robot in the robot’s base coordinate system. $q_{i}={\left[q_{1i},q_{2i},q_{3i}\right]}^{T}$ are the coordinates of a point on the axis of the ith th joint of the robot, typically taken as the origin of that joint coordinate system. $\theta_{i}$ is the rotation angle value of the ith joint of the robot. ${\hat{\xi }}_{i}$ is the joint screw fixed on the ith joint, represented as:

(3) $${\hat{\xi }}_{i}=\left[\begin{array}{cc} {\hat{\omega }}_{i} & V_{i}\\ 0 & 0 \end{array}\right]$$

where ${\hat{\omega }}_{i}\in so(3)$ is an antisymmetric matrix and can be represented as:

(4) $${\hat{\omega }}_{i}=\left[\begin{array}{ccc} 0 & -\omega_{3i} & \omega_{2i}\\ \omega_{3i} & 0 & -\omega_{1i}\\ -\omega_{2i} & \omega_{1i} & 0 \end{array}\right]$$

Since $\omega_{i}$ is the direction vector of each joint axis in the base coordinate system, it is known that $\left\|\omega_{i}\right\|=1$. Therefore, the representation of $e^{{\hat{\omega }}_{i}\theta_{i}}$ can be obtained as:

(5) $$e^{{\hat{\omega }}_{i}\theta_{i}}=I+{\hat{\omega }}_{i}\theta_{i}+\frac{{\left({\hat{\omega }}_{i}\theta_{i}\right)}^{2}}{2!}+\frac{{\left({\hat{\omega }}_{i}\theta_{i}\right)}^{2}}{3!}+\cdots$$

Since ${\hat{\omega }}_{i}$ is an antisymmetric matrix, according to the definition, it holds:

(6) $${{\hat{\omega }}_{i}}^{2}={\hat{\omega }}_{i}{{\hat{\omega }}_{i}}^{T}-{\left\|{\hat{\omega }}_{i}\right\|}^{2}I$$

(7) $${{\hat{\omega }}_{i}}^{3}=-{\left\|{\hat{\omega }}_{i}\right\|}^{2}{\hat{\omega }}_{i}$$

It can be obtained:

(8) $$e^{{\hat{\omega }}_{i}\theta_{i}}=I+{\hat{\omega }}_{i}\sin\left(\theta_{i}\right)+{{\hat{\omega }}_{i}}^{2}(1-\cos(\theta_{i}))$$

Finally, the relationship between the end-effector coordinate system on the rigid body and the base coordinate system can be represented by $g_{st}(\theta_{i})$, with $g_{st}\left(0\right)$ defined as the initial pose of the rigid body in the base coordinate system. Therefore, the rotational motion of the rigid body relative to the fixed axis can be expressed as:

(9) $$g_{st}\left(\theta_{i}\right)=e^{{\hat{\xi }}_{i}\theta_{i}}g_{st}\left(0\right)$$

For the UR10 robot, based on the derivation above, the kinematic model of the robot can be established as:

(10) $$g\left(\theta \right)=e^{{\hat{\xi }}_{1}\theta_{1}}e^{{\hat{\xi }}_{2}\theta_{2}}\ldots e^{{\hat{\xi }}_{6}\theta_{6}}g_{st}\left(0\right)=\left[\begin{array}{cc} R(\theta ) & p(\theta )\\ 0_{1*3} & 1 \end{array}\right]$$

In the equation above, $R(\theta )\in R^{3*3}$ and $p(\theta )\in R^{3*1}$ represent the attitude rotation matrix and the position translation vector, respectively. The orientation and displacement of the robot’s end-effector coordinate system relative to the robot’s base coordinate system is indicated. The POE model ultimately results in a pose transformation matrix.

### 2.2. Robot Calibration Model

#### 2.2.1. Error Calibration Model Based on Spatial Single Point

Considering that errors in the structure and design parameters of the robot are inevitable and have a significant impact on the end-effector positioning accuracy of the robot, it is necessary to calibrate the structural parameter errors of the robot.

In an ideal scenario, the relationship between the end-effector position coordinates and the various parameters of the robot can be represented as:

(11) $$p=\left[\begin{array}{c} p_{x}\\ p_{y}\\ p_{z} \end{array}\right]=f\left(\theta ;\varepsilon \right)$$

in the equation above, $\varepsilon ={\left[\varepsilon_{1},\varepsilon_{2},\ldots ,\varepsilon_{n}\right]}^{T}$ is the parameter vector consisting of the *n* structural parameters to be calibrated for the robot, and $\theta ={\left[\theta_{1},\theta_{2},\ldots ,\theta_{6}\right]}^{T}$ is the vector of angle values for the robot’s six joints. The values of θ are typically collected through experiments beforehand. For the sake of derivation, θ can be treated as a fixed value, and Equation (11) can be rewritten as:

(12) $$p=\left[\begin{array}{c} p_{x}\\ p_{y}\\ p_{z} \end{array}\right]=f\left(\varepsilon \right)$$

Now, assuming there is an error between the nominal values and the actual values of each structural parameter, $\varepsilon ={\left[\Delta \varepsilon_{1},\Delta \varepsilon_{2},\ldots ,\Delta \varepsilon_{n}\right]}^{T}$, the actual value of the robot’s end-effector position can be expressed as:

(13) $$p^{\prime }=\left[\begin{array}{c} p_{x}^{\prime }\\ p_{y}^{\prime }\\ p_{z}^{\prime } \end{array}\right]=f\left(\varepsilon +\Delta \varepsilon \right)$$

Generally, since the errors in the structural parameters are relatively small, a Taylor expansion of Equation (13) around $\varepsilon ={\left[\varepsilon_{1},\varepsilon_{2},\ldots ,\varepsilon_{n}\right]}^{T}$ can be performed and the terms of quadratic order and higher can be neglected. This leads to the error in the robot’s end-effector position as:

(14) $$\Delta p=p^{\prime }-p=\left[\begin{array}{c} {\Delta p}_{x}\\ {\Delta p}_{y}\\ {\Delta p}_{z} \end{array}\right]=\sum_{i=1}^{n}\frac{\partial f}{\partial \varepsilon_{i}}\Delta \varepsilon_{i}=J\Delta \varepsilon$$

The Jacobian matrix J in Equation (14) is represented as:

(15) $$J=\left[\begin{array}{c} \frac{\partial p_{x}}{\partial \varepsilon_{1}}\;\frac{\partial p_{x}}{\partial \varepsilon_{2}}\;\cdots \;\frac{\partial p_{x}}{\partial \varepsilon_{n}}\\ \frac{\partial p_{y}}{\partial \varepsilon_{1}}\;\frac{\partial p_{y}}{\partial \varepsilon_{2}}\;\cdots \;\frac{\partial p_{y}}{\partial \varepsilon_{n}}\\ \frac{\partial p_{z}}{\partial \varepsilon_{1}}\;\frac{\partial p_{z}}{\partial \varepsilon_{2}}\;\cdots \;\frac{\partial p_{z}}{\partial \varepsilon_{n}} \end{array}\right]$$

Structural parameters of robots could be classified into independent parameters and redundant parameters. The redundant parameters lead to singularity in the Jacobian matrix, thereby reducing the robustness of parameter identification and affecting the final calibration results. Therefore, it is necessary to identify and eliminate the redundant parameters of the robots. Correlation coefficient theory has been introduced for identification [27,28]. For two N-dimensional vectors, X and Y, their correlation coefficient is defined as:

(16) $$r=\frac{\sum XY-\frac{\sum X\sum Y}{N}}{\sqrt{\left(\sum X^{2}-\frac{{\left(\sum X\right)}^{2}}{N})(\sum Y^{2}-\frac{{\left(\sum Y\right)}^{2}}{N}\right)}}$$

When $\left|r\right|=1$, it indicates that X and Y are perfectly linearly correlated, while $\left|r\right|=0$ implies that X and Y are linearly independent.

For three or more vectors, the complex correlation coefficient could be calculated to assess their linear relationships. To determine the linear relationship between vector Y and vectors $X_{1}$, $X_{2}$,…, and $X_{k}$, a multivariate linear regression is first performed on vectors Y and $X_{1}$, $X_{2}$,…, and $X_{k}$ to obtain:

(17) $$\hat{Y}={\hat{\beta }}_{0}+{\hat{\beta }}_{1}X_{1}+{\hat{\beta }}_{2}X_{2}+\cdots +{\hat{\beta }}_{k}X_{k}$$

It is important to note that when conducting a complex correlation analysis on the Jacobian matrix, the presence of a constant term is not allowed. Therefore, the above equation needs to be modified as:

(18) $$\hat{Y}={\hat{\beta }}_{1}X_{1}+{\hat{\beta }}_{2}X_{2}+\cdots +{\hat{\beta }}_{k}X_{k}$$

Then, calculate the correlation coefficient between $\hat{Y}$ and Y to obtain the complex correlation coefficient. The formula for calculating the complex correlation coefficient is:

(19) $$R=\frac{\sum \left(Y-\bar{Y})(\hat{Y}-\bar{Y}\right)}{\sqrt{\sum {\left(Y-\bar{Y}\right)}^{2}\sum {\left(\hat{Y}-\bar{Y}\right)}^{2}}}$$

where $\bar{Y}$ represents the arithmetic mean of Y. The complex correlation coefficient R ranges from [0,1]. A larger R indicates a higher degree of correlation between vector Y and vectors $X_{1}$, $X_{2}$,…, and $X_{k}$.

Here are the steps for removing redundant parameters:

(1) Solve the Jacobian matrix *J*_initial_ in the robot calibration model.
(2) Identify columns in *J*_initial_ that are entirely composed of zeros, which are columns that do not contribute to parameter identification in the current calibration model. Remove these columns to obtain the modified Jacobian matrix *J*′.
(3) Calculate the correlation coefficients between columns in *J*′. If |*r*| = 1 or approaches 1, it indicates a linear correlation between the two columns. One column should be removed in such cases to obtain the final Jacobian matrix *J*″.
(4) Calculate the complex correlation coefficients between each column in *J*″ and the other columns. If |*R*| = 1 or approaches 1, it suggests a linear relationship between the current column and some other columns. Remove the current column to derive the final Jacobian matrix, denoted as J.

#### 2.2.2. Pose Transformation Matrix Based on Generalized Error Matrix

In Section 2.2.1, the error calibration model for the robot based on spatial single point is introduced. Using this model for calibration requires knowing the nominal and actual values of the robot’s end-effector position coordinates. The nominal values of the robot’s end-effector position coordinates can be calculated using the angle θ of the robot’s six joints through Equation (11). In this study, the actual values of the robot’s end-effector position coordinates can be obtained via a laser tracker. However, the measured coordinates provided by the laser tracker are represented in its own measurement coordinate system. To perform calibration, it is necessary to unify the measured coordinates and the robot’s end-effector coordinates in the same coordinate system. Therefore, it is essential to calculate the pose transformation matrix between the robot’s base coordinate system and the laser tracker.

The pose transformation matrix in the ideal state is represented as $T_{ideal}$. If the errors in the robot’s structural parameters are neglected, the equation can be written as:

(20) $$\left[\begin{array}{c} \begin{array}{c} x_{measure}\\ y_{measure} \end{array}\\ \begin{array}{c} z_{measure}\\ 1 \end{array} \end{array}\right]=T_{ideal}\cdot \left[\begin{array}{c} \begin{array}{c} x_{ideal}\\ y_{ideal} \end{array}\\ \begin{array}{c} z_{ideal}\\ 1 \end{array} \end{array}\right]=\left[\begin{array}{cc} \begin{array}{cc} n_{x} & o_{x}\\ n_{y} & o_{y} \end{array} & \begin{array}{cc} a_{x} & m_{x}\\ a_{y} & m_{y} \end{array}\\ \begin{array}{cc} n_{z} & o_{z}\\ 0 & 0 \end{array} & \begin{array}{cc} a_{z} & m_{z}\\ 0 & 1 \end{array} \end{array}\right]\cdot \left[\begin{array}{c} \begin{array}{c} x_{ideal}\\ y_{ideal} \end{array}\\ \begin{array}{c} z_{ideal}\\ 1 \end{array} \end{array}\right]$$

If the nominal and actual values of the end-effector positions of the robot are measured in space for M points, the following can be listed:

(21) $$AX=B=\left[\begin{array}{ccc} \begin{array}{c} \begin{array}{c} x_{measure}\left(1\right)\\ x_{measure}\left(2\right) \end{array}\\ \begin{array}{c} \vdots \\ x_{measure}\left(M\right) \end{array} \end{array} & \begin{array}{c} \begin{array}{c} y_{measure}\left(1\right)\\ y_{measure}\left(2\right) \end{array}\\ \begin{array}{c} \vdots \\ y_{measure}\left(M\right) \end{array} \end{array} & \begin{array}{c} \begin{array}{c} z_{measure}\left(1\right)\\ z_{measure}\left(2\right) \end{array}\\ \begin{array}{c} \vdots \\ z_{measure}\left(M\right) \end{array} \end{array} \end{array}\right]\;=\left[\begin{array}{cc} \begin{array}{cc} x_{ideal}\left(1\right) & y_{ideal}\left(1\right)\\ x_{ideal}\left(2\right) & y_{ideal}\left(2\right) \end{array} & \begin{array}{cc} z_{ideal}\left(1\right) & 1\\ z_{ideal}\left(2\right) & 1 \end{array}\\ \begin{array}{cc} \vdots & \vdots \\ x_{ideal}\left(M\right) & y_{ideal}\left(M\right) \end{array} & \begin{array}{cc} \vdots & \vdots \\ z_{ideal}\left(M\right) & 1 \end{array} \end{array}\right]\left[\begin{array}{ccc} \begin{array}{c} \begin{array}{c} n_{x}\\ o_{x} \end{array}\\ \begin{array}{c} a_{x}\\ m_{x} \end{array} \end{array} & \begin{array}{c} \begin{array}{c} n_{y}\\ o_{y} \end{array}\\ \begin{array}{c} a_{y}\\ m_{y} \end{array} \end{array} & \begin{array}{c} \begin{array}{c} n_{z}\\ o_{z} \end{array}\\ \begin{array}{c} a_{z}\\ m_{z} \end{array} \end{array} \end{array}\right]$$

According to the least squares method, it can be obtained:

(22) $$X={\left(A^{T}A\right)}^{-1}A^{T}B=\left[\begin{array}{ccc} \begin{array}{c} \begin{array}{c} n_{x}\\ o_{x} \end{array}\\ \begin{array}{c} a_{x}\\ m_{x} \end{array} \end{array} & \begin{array}{c} \begin{array}{c} n_{y}\\ o_{y} \end{array}\\ \begin{array}{c} a_{y}\\ m_{y} \end{array} \end{array} & \begin{array}{c} \begin{array}{c} n_{z}\\ o_{z} \end{array}\\ \begin{array}{c} a_{z}\\ m_{z} \end{array} \end{array} \end{array}\right]$$

**Figure 2:**
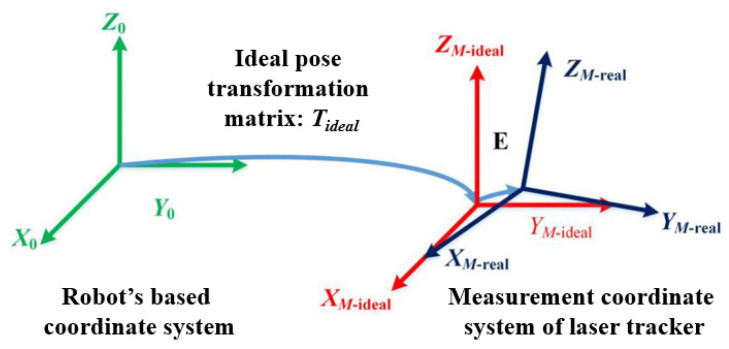
Transformation of coordinate system.

At this point, the computed $T_{ideal}$ does not take into account the errors in the robot’s structural parameters. If the impact of structural parameter errors on the end-effector position error of the robot is considered, there will be a deviation between $T_{ideal}$ and the actual pose transformation matrix, as shown in Figure 2. 

The generalized error matrix is an effective method for describing the error in the pose transformation matrix [22]. By using the generalized error matrix, the relationship between the two systems is described. Assuming the actual pose transformation matrix is $T_{real}$, the relationship between $T_{ideal}$ and $T_{real}$ can be expressed using the generalized error matrix E as:

(23) $$E=Rot\left(X,\tau_{4}\right)Rot\left(Y,\tau_{5}\right)Rot\left(Z,\tau_{6}\right)Trans\left(\tau_{1},\tau_{2},\tau_{3}\right)$$

**Figure 3:**
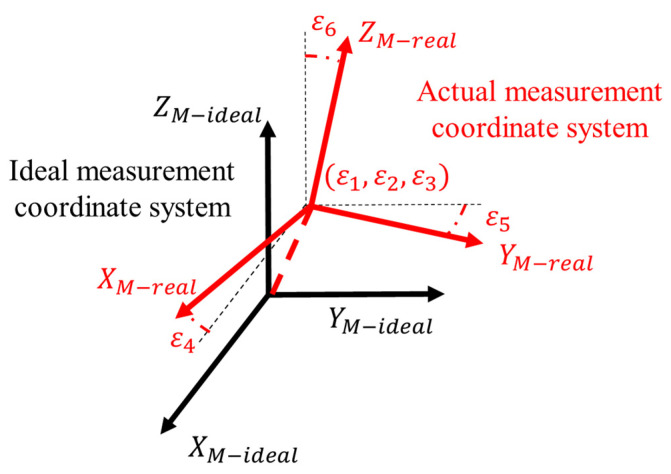
Transformation from the ideal measurement coordinate system to the actual measurement coordinate system.

In Equation (23), $\tau_{4}$, $\tau_{5}$, and $\tau_{6}$ represent the Euler angles of the actual measurement coordinate system relative to the ideal measurement coordinate system, while $\tau_{1}$, $\tau_{2}$, and $\tau_{3}$ represent the displacement of the actual measurement coordinate system relative to the ideal measurement coordinate system, as shown in Figure 3.

Therefore, the position coordinates of the robot’s actual end-effector point in the actual measurement coordinate system can be expressed as:

(24) $$p_{real}=T_{ideal}E\left[\begin{array}{c} p\\ 1 \end{array}\right]={\left[x_{real},y_{real},z_{real},1\right]}^{T}$$

The robot’s structural parameters at this point include the parameters from the generalized error matrix, $\tau ={\left[\tau_{1},\ldots ,\tau_{6}\right]}^{T}$. The error calibration model at this stage is modified to:

(25) $$\Delta p=J\Delta \varepsilon =\left[\begin{array}{cc} \begin{array}{c} \frac{\partial p_{x}}{\partial \varepsilon_{1}}\frac{\partial p_{x}}{\partial \varepsilon_{2}}\cdots \frac{\partial p_{x}}{\partial \varepsilon_{n}}\\ \frac{\partial p_{y}}{\partial \varepsilon_{1}}\frac{\partial p_{y}}{\partial \varepsilon_{2}}\cdots \frac{\partial p_{y}}{\partial \varepsilon_{n}}\\ \frac{\partial p_{z}}{\partial \varepsilon_{1}}\frac{\partial p_{z}}{\partial \varepsilon_{2}}\cdots \frac{\partial p_{z}}{\partial \varepsilon_{n}} \end{array} & \begin{array}{ccc} \begin{array}{c} \frac{\partial p_{x}}{\partial \tau_{1}}\\ \frac{\partial p_{y}}{\partial \tau_{1}}\\ \frac{\partial p_{z}}{\partial \tau_{1}} \end{array} & \begin{array}{c} \cdots \\ \cdots \\ \cdots \end{array} & \begin{array}{c} \frac{\partial p_{x}}{\partial \tau_{6}}\\ \frac{\partial p_{y}}{\partial \tau_{6}}\\ \frac{\partial p_{z}}{\partial \tau_{6}} \end{array} \end{array} \end{array}\right]\Delta \varepsilon$$

(26) $$e_{i}=\sqrt{\begin{array}{c} {\left(x_{real}\left(i\right)-x_{measure}\left(i\right)\right)}^{2}+{\left(y_{real}\left(i\right)-y_{measure}\left(i\right)\right)}^{2}\\ +{\left(z_{real}\left(i\right)-z_{measure}\left(i\right)\right)}^{2} \end{array}}$$

To obtain the vector of errors in the robot’s structural parameters, the calibration problem of the robot can be described as the positioning error of the robot’s end-effector at M points in space. According to the principle of least squares, it can be expressed as:

(27) $$Q=\sum_{i=1}^{M}{e_{i}}^{2}=f\left(\Theta ,P_{measure},\varepsilon ,\Delta \varepsilon \right)$$

Θ and $P_{measure}$ represent the set of robot joint angle values and the corresponding actual end-effector position coordinates used for calibration. Clearly, this optimization problem is a type of nonlinear least squares problem. The Levenberg–Marquardt (LM) method can be used to optimize this problem and obtain the optimized vectors of structural parameter errors.

## 3. Calibration Sampling Data Optimization Method

This section primarily discusses the optimization methods for robot calibration sampling data. In Section 3.1, various observability indices for robots are defined, including $O_{1}$, $O_{3}$, and $O_{d}$. These three observability indices are normalized and combined to form a new observability index $O_{c}$. In Section 3.2, a Binary Simulated Annealing Algorithm introduced in this research is elaborated upon to enhance the quality of the data search in the context of robot calibration.

### 3.1. Establishment of Comprehensive Observability Metrics

When calibrating a robot, people always hope that the end-effector pose errors corresponding to the robot poses used for calibration can sufficiently reflect the errors in the robot’s kinematic parameters in order to obtain more accurate parameter identification results. The magnitude of this reflection capability is essentially the observability of the structural parameter errors by the current robot poses, which can be evaluated using robot observability metrics. Various observability metrics have been proposed.

Assuming that m sets of calibration sampling data for the robot have been obtained, the Jacobian matrix $J_{op}$ can be calculated based on the formula in Equation (15), resulting in a $3m*n_{op}$ matrix, where $n_{op}$ is the number of parameters to be calibrated. Performing a singular value decomposition on $J_{op}$ yields:

(28) $$J_{op}=U\Sigma V$$

In Equation (28), Σ represents:

(29) $$\Sigma =\left[\begin{array}{c} \begin{array}{cc} \begin{array}{cc} \begin{array}{c} \sigma_{1}\\ \vdots \\ 0 \end{array} & \begin{array}{c} 0\\ \vdots \\ 0 \end{array} \end{array} & \begin{array}{cc} \begin{array}{c} 0\\ \vdots \\ \cdots \end{array} & \begin{array}{c} 0\\ \vdots \\ \sigma_{n_{op}} \end{array} \end{array} \end{array}\\ \begin{array}{cc} \begin{array}{cc} \begin{array}{c} 0\\ \vdots \\ 0 \end{array} & \begin{array}{c} 0\\ \vdots \\ 0 \end{array} \end{array} & \begin{array}{cc} \begin{array}{c} \cdots \\ \vdots \\ \cdots \end{array} & \begin{array}{c} 0\\ \vdots \\ 0 \end{array} \end{array} \end{array} \end{array}\right]$$

In Equation (29), $\sigma_{1}$, $\sigma_{2}$, …, $\sigma_{n_{op}}$ are the singular values of $J_{op}$, where $\sigma_{1}\geq \sigma_{2}\geq \cdots \geq \sigma_{n_{op}}\geq 0$.

Borm and Menq [4] introduced the observability metric $O_{1}$:

(30) O1=σ1σ2…σnop1nopm

Driels and Pathre [5] proposed the observability metric $O_{2}$:

(31) $$O_{2}=\frac{\sigma_{n_{op}}}{\sigma_{1}}$$

Nahvi and Hollerbach introduced the observability metric $O_{3}$, also known as the minimum singular value [6]:

(32) $$O_{3}=\sigma_{n_{op}}$$

The observability metrics mentioned above are all aimed at maximizing their respective indicators during the optimization of calibration sampling data. In theory, the larger the observability metric, the better the observability of the errors in the robot’s structural parameters by the robot poses corresponding to the calibration sampling data being used.

#### 3.1.1. The Global Observability Metric $O_{d}$

In addition to observability, another aspect that needs to be considered is the global nature of the robot’s end-effector positions corresponding to the poses. This is because after completing the identification of the robot parameters, to verify the accuracy of the robot calibration, residual end-effector positions (or poses), also known as calibration residuals, are calculated using the calibrated robot parameters under different poses.

Typically, when validating the accuracy of the robot, the closer the poses are during verification to the poses during calibration, the smaller the calibration residuals, and the farther away they are, the larger the calibration residuals. This is because the essence of robot calibration is to use calibration poses and their corresponding end-effector positions as sampling points and approximate the actual values of the robot parameters through a mathematical model.

Based on the analysis above, in order to improve the accuracy of the robot at various poses and enhance the calibration effectiveness, it is necessary to consider the global nature of the robot’s end-effector positions.

Assuming that *m* sets of calibration sampling data for the robot have been obtained, the calculation process for the global observability metric $O_{d}$ is as follows:

(1) Set a value for K.
(2) Compute the Euclidean distance between each pair of the m coordinate points and store them in a matrix D, where D(i,j) represents the Euclidean distance between the *i*th point and the *j*th point.
(3) Sort the elements in each row of ***D*** in ascending order, resulting in the sorted matrix D(i,j).
(4) Calculate the average of the elements in columns 2 to K+1 of each row in $D_{sort}$, i.e., compute the average distance of each point to its nearest K neighbors. This distance is called the K-nearest neighbor distance.
(5) Calculate the variance of the K-nearest neighbor distances, denoted as ${var}_{d}$.
(6) Obtain the global observability metric $O_{d}$ as:

(33) $$O_{d}=\frac{1}{var_{d}}$$

A larger $O_{d}$ indicates a more uniform distribution of the m points in space, signifying better global characteristics.

#### 3.1.2. The Comprehensive Observability Metric $O_{c}$

In practice, robot calibration requires a set of robot poses with optimal comprehensive capabilities and their corresponding end-effector positions to identify kinematic parameter errors. Building upon the previous discussion, a comprehensive observability metric that combines the observability metrics $O_{1}$, $O_{3}$, and $O_{d}$ is proposed to evaluate calibration sampling data in this paper.

When an optimization problem has multiple objectives, the sub-objective functions of multi-objective optimization are typically transformed into a single objective function through mathematical manipulation. A single-objective optimization method is then used to solve the optimization problem. This paper addresses the handling of the three evaluation metrics as follows.

Since the ranges of $O_{1}$, $O_{3}$, and $O_{d}$ are different, the most common normalization method is linear normalization. However, this method requires knowledge of the ranges of the variables being normalized, which is difficult to determine for $O_{1}$, $O_{3}$, and $O_{d}$. Therefore, this study utilizes a normalization method based on the arctangent function:

(34) $$x_{norm}=\frac{2}{\pi }*\mathrm{atan}\left(x\right)$$

It is a form of nonlinear normalization method that utilizes the arctangent function to map the normalized variables to a unified interval. Since all of the variables being normalized are positive, after being processed according to Equation (34), each variable is transformed to the interval [0,1], regardless of its original value. Therefore, the proposed comprehensive observability metric $O_{c}$ is defined as:

(35) $$O_{c}=\frac{2}{\pi }*\left(\mathrm{atan}\left(O_{1}\right)+\mathrm{atan}\left(O_{3}\right)+\mathrm{atan}\left(O_{d}\right)\right)$$

### 3.2. Binary Simulated Annealing Algorithm (BSAA)

In 1953, N. Metropolis et al. introduced the Simulated Annealing Algorithm by simulating the states of individual metal particles during the process of metal annealing. Due to the physical analogy behind the algorithm and its resemblance to combinatorial optimization problems, this algorithm has been widely applied in various fields to solve optimization problems. Compared to other metaheuristic algorithms, the Simulated Annealing Algorithm boasts strong global convergence capabilities, making it less likely to become trapped in local optima. Moreover, this algorithm is characterized by its simple computational structure, versatility, and robustness, making it suitable for optimizing calibration sampling points in robot calibration.

The process of the Simulated Annealing Algorithm can be described as follows: Starting from a relatively high initial temperature, the temperature decreases gradually. At each temperature level, the algorithm performs multiple optimizations in the solution space based on the Metropolis criterion. Even if the next solution found is superior to the current one, there is a certain probability of transitioning to a poorer solution, thus avoiding the issue of local optima. This process continues with temperature reduction and optimization until reaching the set minimum temperature.

**Figure 4:**
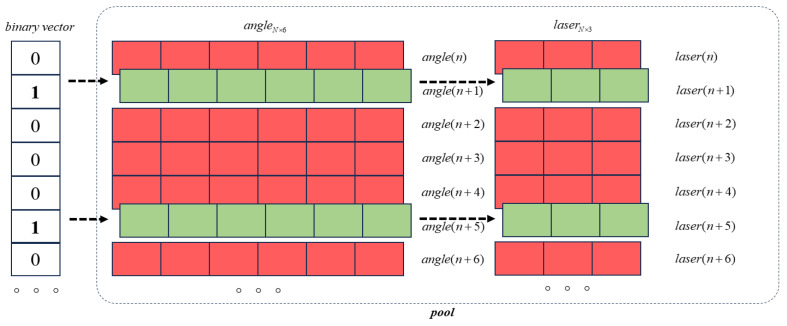
Binary Simulated Annealing Algorithm.

In this study, assuming *m* sets of calibration sampling data for the robot have been obtained, the optimization of calibration sampling data involves finding the optimal data based on observability metrics within these *m* sets of data. Since these data include the joint angles of the robot and their corresponding end-effector coordinates, this optimization process is not a simple low-dimensional problem. Considering that the selection of calibration sampling data have only two states—“yes” or “no”, similar to “0” and “1” in binary—a Binary Simulated Annealing Algorithm suited for selecting optimal robot sampling points is proposed, as shown in Figure 4. The algorithm steps are as follows:

(1) Initialization parameters: Set optimization selection data size $m_{op}$; candidate angle data pool ool=[angle,laser], where angle is an m∗6 matrix storing joint angle data for six different postures, and laser is an m∗3 matrix storing end-effector position coordinates obtained from laser tracker measurements corresponding to angle; set initial temperature $T_{init}=10^{20}$, final temperature $T_{final}=10^{-50}$, cooling rate $R_{cool}=0.98$, and the Markov chain default to 1.
(2) Generate the initial solution and compute the objective function: Randomly generate a binary vector $S_{c}$ composed of $m_{op}$ ones (indicating selected data) and $m-m_{op}$ zeros (indicating unselected data); consider the current best solution $S_{best}=S_{c}$ and compute the corresponding fitness $F=-O_{c}$, i.e., the objective function; and record the current temperature $T_{c}=T_{init}$.
(3) Generate a new solution and compute the objective function, then decide whether to accept the new solution. To generate a new solution $S_{new}$ from the current solution $S_{c}$, perform the following operation: select two positions in $S_{c}$ to flip (changing 1 s to 0 s and 0 s to 1 s). One position is randomly selected from all of the elements that are currently 1, and the other position is randomly selected from all of the elements that are currently 0. After flipping, assign the new vector to $S_{new}$. Calculate the fitness of the new solution $F_{new}=-O_{c}$. Then, use the Metropolis criterion to decide whether to accept the new solution.
(4) Cooling down: Perform the cooling operation by multiplying the current temperature by the cooling rate to obtain the new current temperature, i.e., $T_{c}=T_{c}*R_{cool}$. Check if the final temperature has been reached. If it has, output the final result. Otherwise, increment the iteration counter by one and proceed to step 3.

After utilizing the algorithm for optimization, the result obtained is a collection containing multiple robot postures and their corresponding end-effector positions.

## 4. Experiment

This section primarily presents the experimental content of the study. The experiments consist of both simulation and real-world experiments aimed at validating the effectiveness of the methods proposed in this research. In Section 4.1 and Section 4.2, simulation experiments and real-world experiments are conducted to validate the impact of different optimization methods on the accuracy of robot calibration.

### 4.1. Experiment Introduction

**Figure 5:**
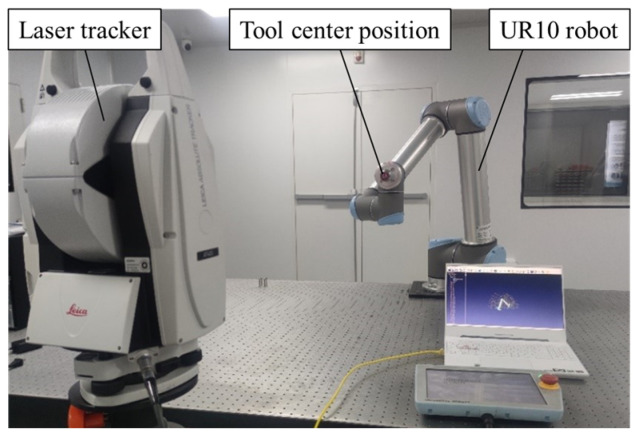
The calibration system of the UR10 robot.

This study is based on the UR10 robot. The experimental system is illustrated in Figure 5.

In the experimental system, the UR10 robot is securely fixed on an optical platform, and its workspace is set, based on laboratory conditions, to be the intersection of the area above the optical platform and the theoretical workspace of the robot. The robot’s control software is RoboDK v4.0.0, which enables communication with the UR10 robot. Users can control the robot to reach a specific point within its workspace through the software interface, where a real-time display is available. The software also provides some interfaces for users to engage in secondary development. The laser tracker Leica AT403 is used, and the maximum single-point measurement error does not exceed 10 μm, meeting the requirements of the experimental research. Considering the laboratory space limitation, working area of the robot, and the characteristics of the laser tracker, the laser tracker is placed approximately 2500 mm to the UR10 robot.

In this calibration experiment, a certain amount of data within the robot’s workspace is collected. There are some positions where the laser tracker cannot track the spherically mounted retroreflector (SMR) because the optical path is obstructed by the robot itself. So partial calibration sampling data in small areas around the positions where the SMR cannot be tracked are collected as the substitute for the missing data. During data collection, after positioning the robot at the target location, the current joint angle data of the robot’s six joints and the end-effector position coordinates measured using the laser tracker are recorded simultaneously. This calibration data are used to compute the ideal pose transformation matrix from the robot’s base coordinate system to the laser tracker measurement coordinate system, and the initial transformation matrix parameters are recorded.

### 4.2. Simulation Experiment

MATLAB R2021a is used to construct a numerical simulation environment to validate the optimization effects of different methods. The theoretical structural parameters of the UR10 robot are obtained from the robot’s manual. Additionally, a small value is added intentionally to the theoretical structural parameters; this value is considered as a structural parameter error. 

In order to set the structural parameter errors in a simulation environment more close to the reality, the structural parameter errors are set according to the average values from the past calibration experiment results. Each component of the direction vectors (corresponding to $\omega_{i}$) and each component of the coordinates (corresponding to $q_{i}$) are within the range [−1.5, 1.5]. This process is conducted to obtain what can be considered as the real structural parameters of the robot in the simulation environment.

Multiple sets of joint angle values for the robot’s six joints are generated to simulate the calibration process in actual experiments. The generated angle values are all within the range of motion for the robot’s joints. The ranges of motion for the six joints are listed in Table 1. For each set of joint angle values generated, the corresponding coordinates are calculated using the model. Random numbers within the range [−0.01, 0.01] are added to the calculated coordinates to simulate the inherent random errors in actual calibration. 

Subsequently, datasets are obtained. Different methods are applied to optimize the calibration sampling data, and the LM method is used to process the optimized parameters.

To validate the optimization effects of different methods, 50 sets of data generated using the same method are used to assess the robot’s positioning error. Assessing the robot’s positioning error could evaluate whether the robot’s parameter errors are close to the actual values. Considering that the DETMAX algorithm is a more traditional method in the realm of sampling point optimization [15], contrasting experiments among different search methods are carried out. Additionally, to verify the effectiveness of the observation metrics proposed, a comparison is made between the $O_{c}$ metric and the $O_{1}$, $O_{2}$, and $O_{3}$ observation metrics. The relevant statistical results are shown in Table 2.

It is found that calibrating the robot using the data optimized by combining BSAA with $O_{c}$ resulted in lower average positioning errors and root mean square errors compared to other methods. When the search algorithm remained the same, using the $O_{c}$ observation metric yields slightly better results than using other observation metrics; similarly, when the observation metric remained the same, the results obtained with the BSAA search algorithm are better than those obtained with the DETMAX algorithm. Therefore, in the simulation environment, the calibration sampling data optimization method proposed in this study holds a greater advantage compared to other methods.

### 4.3. Calibration Experiment

**Figure 6:**
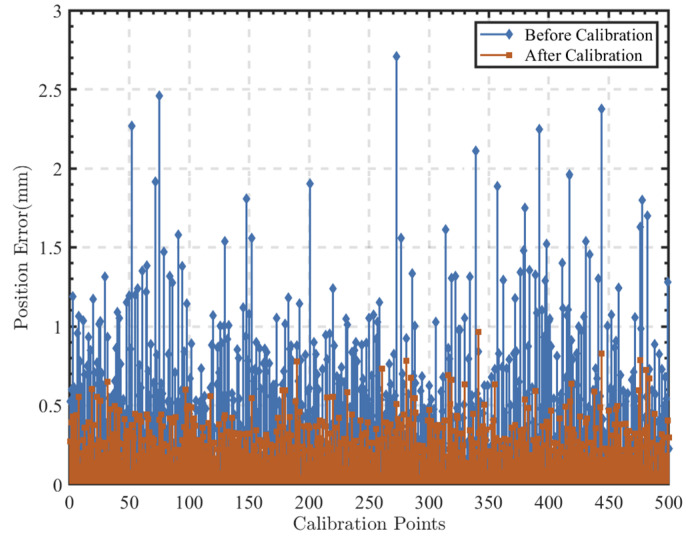
Comparison of the positioning error for the sampling points at the calibration.

**Figure 7:**
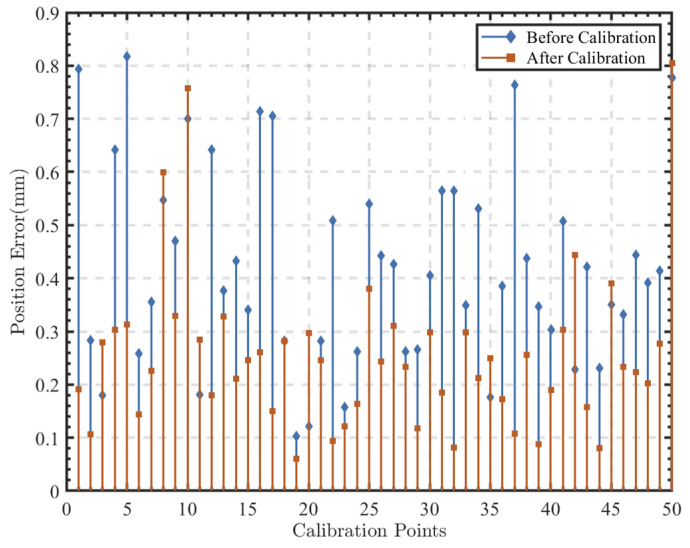
Comparison of the positioning error for the verification points at the calibration.

In total, 583 calibration sampling data points within the workspace of the robot are collected. The saved sampling point data underwent point optimization as described earlier, where 500 optimized sampling points are selected from all of the data as the final calibration data. The structural parameters obtained through optimization using the Levenberg–Marquardt method are shown in Table 3, and the end-effector positioning errors before and after optimization are depicted in Figure 6. To validate the compensation effect, data from 50 randomly sampling points within the robot’s workspace are collected, and the end-effector positioning errors of the robot are shown in Figure 7.

To validate the effects of different methods, a comparative experiment similar to the simulation experiment was conducted. The data optimized using different methods were used for robot calibration, and 50 positions within the workspace were selected for positional accuracy verification. The relevant statistical results are shown in Table 4. 

It is observed that optimizing the calibration sampling data using observation metrics can improve the end-effector positioning accuracy of the robot. By optimizing the calibration sampling data using the BSAA method combined with the $O_{c}$ observation metric, the end-effector positioning accuracy improves from 0.356 mm to 0.254 mm after calibration, representing an overall enhancement of 28.58% compared to not using optimization methods.

Analyzing the results of other optimization methods, it is found that when the search algorithm is the same, the average positioning accuracy and root mean square error obtained after optimizing with the $O_{c}$ observation metric are better than those obtained with other observation metrics. Similarly, when the observation metric is the same, the BSAA proposed in this study achieves better robot positioning accuracy compared to the traditional DETMAX algorithm.

These conclusions align closely with the findings from the simulation experiments. However, whether in simulation or real-world experiments, the maximum value of robot positioning accuracy seems to have no direct correlation with the search algorithm or observation metrics used. Overall, the method proposed in this study remains effective in improving the results of robot calibration.

## 5. Discussion

In this study, a new method for optimizing robot sampling points is proposed. The optimized sampling points obtained using this method can enhance the calibration quality of the robot, leading to more accurate robot kinematic parameters and improving end-effector positioning accuracy compared to the previous method. This is because the previous observability metrics, *O*_1_, *O*_2_, *O*_3_, *O*_4_, and *O*_5_, are all aimed at maximizing their respective indicators during the optimization of calibration sampling data to reduce the influence of non-kinematic factors, and these observability metrics do not reflect the uniformity of sampling point coverage, while the global observability metric *O*_d_ proposed in this paper could improve the uniformity. And only the observability metrics *O*_1_ and *O*_3_ have little correlation and could be evaluated independently from two perspectives, so these two observability metrics are selected. Therefore, using the comprehensive observability metric *O_c_* has the advantage of reducing the influence of non-kinematic factors and improving the sampling uniformity. And the BSAA has the advantages of overcoming the local optimization. Initially, a robot kinematic model based on the POE model and the generalized error model is established. The ideal pose transformation relationship between the robot base coordinate system and the laser tracker measurement coordinate system is derived using the least squares method to create a spatial single-point error calibration model. Subsequently, calibration sampling data are collected within the robot’s workspace. An improved robot observation metric, combined with the Binary Simulated Annealing Algorithm proposed in this study, is employed for optimizing the calibration sampling data. Finally, the robot’s parameter error vector is obtained using nonlinear least squares methods.

The average end-effector error of the robot is reduced from 0.356 mm to 0.254 mm by the use of sampling points obtained using the proposed method. This improvement indicates an enhancement in calibration quality compared to the previous method.

There is still much work to be carried out in the field of robot calibration techniques. In the future, improvements should be made to the robot’s kinematic model to account for more sources of error, thereby enhancing the model’s accuracy. Additionally, the current method of selecting calibration data involves searching for optimal data within a large set of calibration data. However, this approach is limited by the distribution of the robot within the workspace during the initial data collection.

To address this limitation, it would be beneficial to use a simulation and mathematical analysis to proactively identify optimal measurement positions within the robot’s workspace. This method could help save costs by determining the preferred measurement locations in the robot’s workspace before data collection, rather than relying solely on data gathered during the initial calibration process. And more a suitable simulation settings method needs to be further researched.

Furthermore, during the process of collecting calibration data, due to the fixed positioning of the laser tracker, there will always be regions where the laser tracker cannot track the SMR due to obstruction by the robot itself. As a result, the collected data may not fully reflect the global characteristics of the robot. Research addressing this issue is equally meaningful, and the relative data processing method will be one of the directions for future investigation.

## Figures and Tables

**Table 1:** Range of robot’s joints.

	Joint 1	Joint 2	Joint 3	Joint 4	Joint 5	Joint 6
Range (°)	−363~363	−363~363	−363~363	−363~363	−363~363	−363~363

**Table 2:** Positioning error with different method in simulation experiment.

Method	Mean (mm)	RMSE (mm)	Max (mm)
None	0.485	0.558	1.078
$\mathrm{Detmax}\;\mathrm{with}\;O_{1}$	0.4131	0.4807	1.0966
$\mathrm{Detmax}\;\mathrm{with}\;O_{2}$	0.430	0.491	1.015
$\mathrm{Detmax}\;\mathrm{with}\;O_{3}$	0.421	0.487	1.196
$\mathrm{Detmax}\;\mathrm{with}\;O_{c}$	0.399	0.467	1.201
$\mathrm{BSAA}\;\mathrm{with}\;O_{1}$	0.388	0.457	1.113
$\mathrm{BSAA}\;\mathrm{with}\;O_{2}$	0.399	0.463	1.133
$\mathrm{BSAA}\;\mathrm{with}\;O_{3}$	0.382	0.430	0.832
$\mathrm{BSAA}\;\mathrm{with}\;O_{c}$	0.363	0.411	0.982

**Table 3:** Robot parameter after calibration.

Parameter	Joint 1	Joint 2	Joint 3	Joint 4	Joint 5	Joint 6	Tool
$w_{i1}$	−0.0019	−0.0039	−0.0021	−0.0040	−0.0069	0	0
$w_{i2}$	−0.308	−0.9992	−1.0006	−0.9994	0.0304	−1	−0.00256
$w_{i3}$	0.9999	−0.0302	−0.0259	−0.0228	−1.0004	0	−0.00163
$q_{i1}$	1.9767	0.8579	−613.3492	−1184.42	−1186.48	−1184.34	−1184.30
$q_{i2}$	3.8980	0	0	0	−162.3182	−163.9410	−164.8382
$q_{i3}$	0	129.8632	128.3573	128.0043	127.3000	11.6849	11.6000

**Table 4:** Positioning error with different method.

Method	Mean (mm)	RMSE (mm)	Max (mm)
None	0.356	0.397	0.280
$\mathrm{Detmax}\;\mathrm{with}\;O_{1}$	0.322	0.386	0.312
$\mathrm{Detmax}\;\mathrm{with}\;O_{2}$	0.330	0.351	0.370
$\mathrm{Detmax}\;\mathrm{with}\;O_{3}$	0.326	0.360	0.262
$\mathrm{Detmax}\;\mathrm{with}\;O_{c}$	0.303	0.356	0.267
$\mathrm{BSAA}\;\mathrm{with}\;O_{1}$	0.299	0.356	0.287
$\mathrm{BSAA}\;\mathrm{with}\;O_{2}$	0.327	0.356	0.342
$\mathrm{BSAA}\;\mathrm{with}\;O_{3}$	0.319	0.351	0.175
$\mathrm{BSAA}\;\mathrm{with}\;O_{c}$	0.254	0.293	0.805

## Data Availability

Data available on request due to restrictions, e.g., privacy or ethical.
